# Smartphone-based dispatch of community first responders to out-of-hospital cardiac arrest - statements from an international consensus conference

**DOI:** 10.1186/s13049-021-00841-1

**Published:** 2021-02-01

**Authors:** Camilla Metelmann, Bibiana Metelmann, Dorothea Kohnen, Peter Brinkrolf, Linn Andelius, Bernd W. Böttiger, Roman Burkart, Klaus Hahnenkamp, Mario Krammel, Tore Marks, Michael P. Müller, Stefan Prasse, Remy Stieglis, Bernd Strickmann, Karl Christian Thies

**Affiliations:** 1 Department of Anaesthesiology, University Medicine Greifswald, Ferdinand-Sauerbruch Straße 1, 17489 Greifswald, Germany; 2 zeb.business school, Steinbeis University Berlin, Münster, Germany; 3 Copenhagen Emergency Medical Services, University of Copenhagen, Copenhagen, Denmark; 4 Department of Anaesthesiology and Intensive Care Medicine, University Hospital of Cologne, Cologne, Germany; 5 Ticino Cuore Foundation, Breganzona, Switzerland; 6 Emergency Medical Service Vienna, Vienna, Austria; 7 PULS Austrian Cardiac Arrest Awareness Association, Vienna, Austria; 8 Department of Anaesthesiology, Intensive Care and Emergency Medicine, St. Josefskrankenhaus, Freiburg im Breisgau, Germany; 9 Mobile Retter e.V, Köln, Germany; 10 Department of Cardiology, Amsterdam UMC, University of Amsterdam, Amsterdam, The Netherlands; 11 Emergency Medical Service, City and District of Gütersloh, Gütersloh, Germany; 12 Klinik für Anaesthesiologie, EvKB, Universitätsklinikum OWL der Universitaet Bielefeld, Campus Bielefeld-Bethel, Burgsteig 13, 33617 Bielefeld, Germany

**Keywords:** Resuscitation, Out-of-hospital cardiac arrest, First responders, Citizen responder, Consensus, mHealth, Smartphone

## Abstract

**Background:**

Over the past decade Smartphone-based activation (SBA) of Community First Responders (CFR) to out-of-hospital cardiac arrests (OHCA) has gained much attention and popularity throughout Europe. Various programmes have been established, and interestingly there are considerable differences in technology, responder spectrum and the degree of integration into the prehospital emergency services. It is unclear whether these dissimilarities affect outcome. This paper reviews the current state in five European countries, reveals similarities and controversies, and presents consensus statements generated in an international conference with the intention to support public decision making on future strategies for SBA of CFR.

**Methods:**

In a consensus conference a three-step approach was used: (i) presentation of current research from five European countries; (ii) workshops discussing evidence amongst the audience to generate consensus statements; (iii) anonymous real-time voting applying the modified RAND-UCLA Appropriateness method to adopt or reject the statements. The consensus panel aimed to represent all stakeholders involved in this topic.

**Results:**

While 21 of 25 generated statements gained approval, consensus was only found for 5 of them. One statement was rejected but without consensus. Members of the consensus conference confirmed that CFR save lives. They further acknowledged the crucial role of emergency medical control centres and called for nationwide strategies.

**Conclusions:**

Members of the consensus conference acknowledged that smartphone-based activation of CFR to OHCA saves lives. The statements generated by the consensus conference may assist the public, healthcare services and governments to utilise these systems to their full potential, and direct the research community towards fields that still need to be addressed.

**Supplementary Information:**

The online version contains supplementary material available at 10.1186/s13049-021-00841-1.

## Introduction

Numerous studies have demonstrated an increase in favourable neurological outcome, if cardiopulmonary resuscitation (CPR) commences within the first minutes of cardiac arrest [1]. This cannot be achieved by the Emergency Medical Service (EMS) alone; even if they arrive on scene within 5 min, immediate bystander CPR still leads to a two- to threefold increase in survival [2, 3]. To bridge the time until EMS arrival, various systems worldwide introduced deployment of Community First Responders (CFR) to out-of-hospital cardiac arrests (OHCA) [4–13]. This led to an increased rate of CPR performed before arrival of the EMS [14, 15]. The spectrum of volunteers deployed is ranging from lay persons without medical background to highly trained medical professionals [4, 6, 7, 11–13, 16–18].

CFR dispatch systems initially worked with text messaging, but switched to smartphone-based activation (SBA) via specific smartphone applications (apps) [19, 20]. These apps use georeferencing to detect CFR within a defined radius of the site of an incident and guide them to the scene using a navigation system [21]. The most advanced apps select and engage first responders based on estimated journey time and not on distance. Switching from text messaging to SBA has proven to be highly effective and led to a further decrease in no-flow time [20].

CFR equipped with automated external defibrillators (AED) led to a significant earlier defibrillation and an increased 30-day and also three-year survival in OHCA [11, 15, 22–25]. A Dutch study revealed that dispatch of CFR seems to be particularly beneficial in witnessed OHCA occurring at home during evening hours or at night when EMS response times are above average [26].

Currently, in more than half of the European countries SBA systems can be found in at least a small region [12, 17]. However, these systems differ in several aspects, e.g. technology, qualification of CFR, type of medical emergencies responders attend, and training of CFR [17]. Given the absence of guidelines on SBA of CFR, the existing systems cannot be compared against a standard. It is also not clear yet how the different SBA systems affect outcome of OHCA. This in turn could mean that app systems are not being used to their full potential. Moreover, it would be very helpful for patients, if different systems would be compatible in a way that CFR from one system would be able to respond also in other systems.

Hence, an international consensus conference was held in September 2019 in Greifswald, Germany to share experience and identify common ground amongst the stake holders (providers, scientists, EMS operators, health insurances, government, and the public). The output of this conference may help aligning future strategies for SBA of CFR and encourage research and collaboration.

## Methods

### Consensus panel

The consensus panel consisted of the faculty and the non-faculty participants (attendees) of the meeting. Faculty were recruited by the scientific commitee, who did a literature search to identify and invite research groups working on SBA of CFR. The attendees represented stakeholders with an interest in OHCA, including members of the public, CFR, firefighters, researchers, allied health care professionals, doctors, health care managers, politicians, representatives of the public administration and health insurances, as well as app developers and vendors. To reach all stakeholders more than 2000 personal invitations were sent to regional and national representatives of the public administration and government; to representatives of all major health insurances in Germany; to all EMS dispatch centres in Germany; to non-governmental first aid organisations throughout Germany; to CFR; and to all app developers and vendors in central Europe, which were hand searched by the scientific committee. Additional promotion was spread through a multitude of advertisement posted on the website of all relevant societies and corresponding professional news outlets (e.g. German Society of Anaesthesiology and Intensive Care Medicine; German Resuscitation Council and German Association of Anaesthesiologist, news-papers.eu). To address the public multiple press releases were issued; students at the two local universities were invited; and social media were utilised intensely. While the majority of attendees was from Germany, others travelled from Denmark, The Netherlands, Switzerland and Austria.

All panel members stated their individual conflicts of interests, consented to participate in the consensus process and agreed to publication of the results. The authors of this article were also part of the consensus panel, with the exception of the non-voting member CM.

### Consensus process

The conference was organized by the Department of Anaesthesiology of Greifswald University Medicine under the auspices of the German Resuscitation Council (GRC) and the German Association of Anaesthesiologists (BDA). To find consensus an adaptation of the RAND-UCLA Appropriateness Method [27] was used employing a three-step approach: (i) presentations and discussions on the current state of practice and research, (ii) open break out workshops, where faculty and attendees produced consensus statements, (iii) which were subsequently adopted or rejected following the RAND-UCLA method. RAND-UCLA method was developed to evaluate whether the expected benefits of a medical intervention outweighs the expected harm to such a degree, that implementation should be recommended [27]. In RAND-UCLA Appropriate Method a literature review lays the foundation for experts rating the benefit-to-harm ratio for different aspects of the new intervention on a 9-point scale. A point of 1 assesses the intervention as unsafe as expected harms greatly outweigh expected benefits; while a point of 9 strongly recommends the intervention, because expected benefits greatly exceed expected harm [27].

(i) Presentation of the current state of research

The conference started with presentations from national and international speakers reporting on their experience and research in the field of SBA of CFR. CFR systems in Denmark, the Netherlands, Switzerland, Austria, and Germany were presented and discussed with a focus on their differences and similarities. To facilitate comparison of the systems all speakers were asked to address a set of topics listed in Table 1.

**Table 1:** Comparison between different CFR systems in Europe

Comparison between different CFR systems in Europe: topics addressed during the presentation	
- Qualifications of responder	
- Training	
- Debriefing responders	
- Recruitment and retainment of responders	
- Effect on patient outcome	
- Funding	
- Examples of good practice and learning points	

Additionally, all major CFR app developers were invited to present their systems and attendees of the conference had the opportunity to compare different products. For probity reasons this session was held as a separate industry forum.

Both research and product presentation served as basis for the discussion and generating consensus statements during the open break out workshop sessions.

(ii) Open break out workshops

The subsequent workshop session lasted 40 mins and was held in an open space allowing for six workshops simultaneously addressing different topics, see Table 2. All attendees were invited to take part in the workshops and actively contribute to the creation of the consensus statements. Each workshop was led by two faculty members, who facilitated the discussion, took notes on a whiteboard and ensured that the views of all attendees were reflected in the consensus statements. The attendees could choose freely which workshop to join. There was no set number of attendees per workshop. The attendees were also encouraged to move between workshops during the session to augment exchange of expert knowledge and stakeholder views on the different topics. However, one limitation might be that discussions were interrupted by panel members joining or leaving the workshop.

(iii) Voting

**Table 2:** Workshop topics

Workshop topics	
- Fields of application	
- Qualifications of CFR	
- Education/Training	
- Role of EMS dispatch centres	
- Funding	
- Recruitment and Retaining CFR	

The consensus statements were presented to all participants of the conference and put to vote during stage three of the modified RAND-UCLA process, which started immediately following the break out workshops. To enable anonymous real-time voting, the mobile phone software PINGO (University of Paderborn, Germany) was used. The consensus statements were presented sequentially to all participants of the conference the consensus panel on a screen and on the voting tool. After the statement was presented and read out, it was directly voted on. To allow the participants to familiarize themselves with the technique, voting time for the first and second statement were 1 min respectively. Thereafter voting times for the remaining statements were reduced to 30 s. All participants had the same vote independent from experience and background. In line with the RAND-UCLA Appropriateness Method [27] all statements were assessed with a nine-point scale, with “1” meaning “rejection”, “5” “uncertain” and “9” “approval”. The voting results were later grouped into approval/rejection and consensus was determined based on the criteria listed in Table 3.

**Table 3:** Modified RAND-UCLA classification criteria

Modified RAND-UCLA classification criteria	
Level of agreement	
- Approval: median > 6.5	
- Uncertain: median ≥ 3.5 and ≤ 6.5	
- Rejection: median < 3.5	
Level of consensus	
- High consensus	
° Voting results exclusively in the range of 7 to 9	
° Voting results exclusively in the range of 1 to 3	
- Consensus	
° Mean - standard deviation > 7	
° Mean + standard deviation < 3	
- Trend	
°Mean - standard deviation < 5	
°Mean + standard deviation > 5	
- No consensus	
° Voting results range from 1 to 9	

## Results

A total of 101 participants joined the conference representing a variety of different stakeholders (Table 4), of which 12 were faculty from Germany, Denmark, The Netherlands, Switzerland and Austria.

**Table 4:** Conference attendees representing different stakeholders

Stakeholder group	Number of conference attendees (n)
Public	7
CFR	15
Firefighters	6
Researchers	17
Allied health care professionals	7
Doctors	9
Health care managers	13
Politicians and public administration	14
Health insurance	4
App developers and vendors	9
*Total number of participants*	*101*

Of these 41 joined the consensus panel. During the break out workshops, 24 consensus statements were generated. The statement “CFR save lives” was developed by the scientific committee in advance. All 25 consensus statements were rated by at least 35 of 41 panellists. While 21 of 25 statements gained approval, one statement was rejected. Consensus was found for 20% (5 out of 25 statements). In 11 out of 25 statements answers were given covering the whole range from 1 to 9. Table 5 lists the 25 statements with level of agreement and level of consensus. Detailed voting results of the consensus panel with mean and standard deviation are available as Supplementary Data.

**Table 5:** Level of agreement and level of consensus

Consensus statements	Level of agreement	Level of consensus
CFR save lives.	Approval	High consensus
**Fields of application**		
CFR should be dispatched to OHCA only.	Approval	No consensus
CFR should also be dispatched to children under the age of 8 years.	Approval	No consensus
Persons older than 16 years should be dispatched as CFR.	Approval	No consensus
**Qualifications of CFR**		
Medical laypersons should also be CFR.	Approval	No consensus
It is better to have many unqualified CFR than few but qualified CFR.	Uncertain	No consensus
To become CFR one should know how to perform CPR.	Approval	Trend
**Education/Training**		
Every CFR should train CPR every 2 years.	Approval	Trend
CFR should receive training covering organisational, legal and medical aspects.	Approval	No consensus
Training to become CFR could be e-learning without an actual meeting.	Rejection	No consensus
CFR should be prepared systematically for acute psychological stress.	Approval	No consensus
**Role of EMS dispatch centres**		
Only EMS dispatch centres should be able to dispatch CFR.	Approval	High consensus
The software of the EMS dispatch centre should automatically recommend a CFR.	Approval	Trend
The EMS dispatch centre should be able to contact a CFR during a mission.	Approval	Consensus
CFR should be able to contact the EMS dispatch centre during a mission.	Approval	Consensus
The EMS dispatch centre should be notified about the qualification of the CFR.	Uncertain	No consensus
**Funding**		
CFR-systems should be financed by health insurance funds.	Approval	Trend
CFR-systems should be laid down in the social security code.	Approval	Trend
CFR-systems should be financed on an interim basis within the federal state law.	Approval	Trend
**Recruitment and Retaining CFR**		
We need a nationwide strategy.	Approval	Consensus
CFR should be implemented in the EMS.	Approval	Trend
Participation in the CFR-system should be actively advertised.	Approval	Trend
Potential CFR should be recruited with help of established aid organisations.	Approval	No consensus
All CPR by CFR should be made public.	Uncertain	No consensus
An exchange of experiences should be organised after a certain time.	Approval	Trend

## Discussion

During the conference, the consensus panel had the opportunity to compare different CFR systems implemented in five European countries (Denmark, The Netherlands, Germany, Switzerland, and Austria). Pros and Cons of the different approaches were discussed. The debate and voting reflected the wide variety between the different systems and even opposing opinions in some aspects. Hence, consensus could only be found in 5 out of 25 generated statements.

It was agreed with high consensus, that Community First Responders save lives. This conclusion is supported by an observational study showing an increased survival to hospital discharge, if OHCA was attended by at least one first responder [16].

Approval with high consensus was also found for the statement, that activation of CFR should be done by EMS dispatch centres. Deployment of CFR by the dispatch centres offers better integration into the EMS system [28]. Additionally, the dispatch centre can evaluate for each situation, whether the dispatch of a CFR is safe [29]. In systems working detached from EMS dispatch centres, there is no specific risk assessment of each mission. Some SBA systems allow activation of CFR by both medical laypersons and emergency medical dispatch centres [30].

Consensus was also found on enabling communication between CFR and EMS dispatcher during the mission, with both being able to initiate contact. Studies done in CFR-system working without SBA, showed that some CFR appreciated support during a mission [31] and found lack of information stressful and frustrating [32]. The possibility to contact the dispatch centre to check back or gain further information might alleviate the stress level of CFR.

Consensus was found on the need for a national strategy. Currently, a multitude of successful CFR systems exists worldwide, which differ in several aspects, e.g. technology, training scope, funding and mode of activation of CFR [17]. Even within single countries, varieties of CFR systems exist [7, 17]. This diversity could negatively affect effectiveness, safety and retention of personnel [7]. The high Dutch OHCA survival rates are attributed to a nationwide alert system that dispatches primarily BLS trained laypersons but also professional responders as fire service and police [16]. Switzerland has launched a national project in January 2020 linking cantonal SBA systems with a national mobile app. While domestic CFR are alerted through their own cantonal SBA system, visiting CFR from neighbour cantons are simultaneously engaged through the national app. As a result, CFR registered in one canton no longer need to register in another canton as well. Efforts should be taken to offer such technique also on national and international levels elsewhere.

Diversities between different CFR systems arose due to different backgrounds, geographical and infrastructural conditions. A Swedish study on dispatch of firefighters alerted without smartphone app analysed the impact of population density: In rural areas the relative reduction in median time until first chest compression was greater than in urban areas. However, the increase of 30-day-survival was much higher in densely populated areas [33]. Hence, we might need to adapt CFR systems to differing regional demands [28]. One approach in rural areas could be to build a tighter net by also including medical laypersons as CFR.

The inclusion of medical laypersons as first responders was discussed controversially. The obvious benefit of a tighter net of CFR has to be balanced with the counteracting aspect, that quality of CPR may not be as good in laypersons as in professionals. There are indications that survival rate of OHCA is doubled if bystander CPR is performed by medically trained personnel instead of laypersons [34, 35]. Bystander CPR initiated by medically trained CFR instead of laypersons was associated with higher 30-day-survival [36].

Most members of the consensus panel agreed, that a person needs basic knowledge of CPR to become CFR. Yet, systems successfully implemented in Singapore and Italy are open for laypersons without any training in CPR [13, 37]. In the Italian system CFR get instructions on chest compressions by the dispatch centre whilst attending an OHCA [37].

Training is perceived as fundamental by first responders and some feel that training shows that the organisation values their effort [31]. Most CFR are in favour of scenario-based learning [31, 38]. Accordingly, the consensus panel disapproved of e-learning without face-to-face training.

It’s uncertain which other aspects apart CPR should be addressed during training. A focus should also be put on safety [32]. Because confidentiality is a major concern, legal aspects are recommended [6]. In addition, CFR would also like to receive training in communicational skills and dealing with emotional aspects [5, 7, 31]. Working as CFR can be traumatic and emotionally stressful [5, 6]. It is unclear, if there should be a minimum age to become a CFR. Most systems in Europe operate an age threshold of 16 years [17]. Focused training for CFR could help alleviate the psychological burden. A Dutch study showed, that even if lay rescuers experience severe stress and short-term psychological impact, no symptoms of post-traumatic stress disorder could be detected 4 to 6 weeks afterwards [39].

During the consensus conference, there was an in-depth debate, whether CFR should also attend paediatric OHCA. While some systems also alert to paediatric OHCA, most do not [40]. First responders found CPR on children to be more distressing than on adults [32]*.* Albeit, children might benefit most from early resuscitation by CFR.

It was discussed in great detail, if CFR should be dispatched only to OHCA or to other medical emergencies as well, and no consensus could be found. While most CFR systems dispatched initially only to OHCA, the role of CFR has increased over time [31]. When determining, which emergencies first responder should attend, need for additional training and equipment should be considered. Basic life support requires only minimal equipment, e.g. gloves. CFR can approach the emergency site without needing to collect a special kit. In contrast, treatment of other emergencies requires additional equipment and CFR may need to detour. The major benefit of CFR systems is time advantage in relation to EMS. Taking a detour would impair this. Some systems also include automatic external defibrillators (AED), because a dispatch of first responders equipped with AED was found to lead to earlier shock and increased survival [4, 18]. However, picking up an AED may lead to a delay in CPR [41], especially in areas with low population densities and limited availability of AED. Results from a Dutch study indicate, that the optimum constellation is more than ten First Responders and 2 AED per square kilometre [42].

Funding of CFR programmes differ, even within one country [6]. Systems typically depend on fundraising or receive, to some degree, statutory funding [31]. During the consensus process some participants suggested, that CFR programmes should be laid down in the social security code and financed by health insurance funds or on an interim basis within the federal state law.

One possibility to fund CFR is to implement it into existing EMS structures. Currently, some CFR systems are part of the EMS, while others are complementary or totally separate from the EMS and sometimes they replace EMS (remote areas in Iceland) [9, 17, 43].

A collaboration with well-established non-governmental first aid organisations may be beneficial to promote CFR systems. To recruit new CFR, participation has to be actively promoted and advertised; first responders frequently discover CFR programmes via promotion material or by talking to CFR [10, 13, 31]. Commitment to being a CFR should be actively supported [44].

A way to increase public recognition, would be to announce all resuscitations performed by CFR. In general, the community wants to be better informed about CFR [6]. Additionally, many first responder expect praise for their help [7], which might help to retain CFR.

Regular meetings of the CFR group may also help to increase retention [9]. CFR who have not been dispatched for a longer period may get demotivated [10]. CFR groups with regular meetings were found to have higher cohesion and motivation [10]. Thus, a platform for communication and training is recommended.

It remains unclear whether a system with many unqualified responders delivers better outcomes than a system with a few highly qualified CFR. Hence, in some countries, like UK, systems with a small number of medical professionals coexists with systems with a large number of medical laypersons [9]. Lessons learned from the different systems worldwide might help us answer this question and randomised controlled trials are needed [12, 17, 43].

### Limitations of the study

The chosen format entails a few limitations. The conference was held in German and English, which implies a certain language bias.

Despite our best efforts to engage with all stakeholder groups the participants may not reflect the target population properly. Nevertheless, we managed to recruit a variety of opinion leaders and stakeholders from different backgrounds. Some participants might have had a higher impact on others during the discussion, which we had anticipated and which we have tried to mitigate through the workshop leads, who ensured that all participants were heard during the discussions. The voting process also mitigated the influence of strong opinion leaders. Voting results reflect the opinion of the stakeholders who participated in the voting session.

Transferability of the data may be limited, because focus was laid on CFR systems in Europe. A consensus process condenses complex aspects, which has the potential drawback of overgeneralisation. However, a consensus process offers an opportunity to analyse the current state and identify areas, which need further evaluation. Despite the limitations of the chosen format, this is, to our knowledge, the first consensus process, bringing together stakeholders from various backgrounds and integrating scientific evidence, the views of the public, the political decision makers and the health care budget holders.

## Conclusions

This paper shows the current consensus and divergences regarding implementation of dispatch of CFR to OHCA. While some statements were approved with consensus, others are still open for discussion. Members of the consensus conference acknowledged the integral role of the emergency medical dispatch centre and confirmed that CFR save lives. Hence, nationwide strategies are recommended, to utilise smartphone-based activation of CFR to its full potential.

The consensus conference was held to identify the current state and answer some pending questions. The authors try to encourage further research and collaboration in this field. Smartphone-based activation of CFR to OHCA is a great tool to save more lives. However, it is not clear yet, how to maximise their benefit.

## Supplementary Information

**Additional file 1.**

## Data Availability

Data generated and analysed during this study are included in this published article. Raw data are available from the corresponding author on reasonable request.

## Abbreviations

AED: Automated external defibrillator
ALS: Advanced Life Support
App: Smartphone application
BDA: German Association of Anaesthesiologists
CFR: Community First Responder
CPR: Cardiopulmonary resuscitation
DIVI: German Interdisciplinary Association for Intensive and Emergency Medicine
EMS: Emergency Medical Service
ERC: European Resuscitation Council
GRC: German Resuscitation Council
ILCOR: International Liaison Committee on Resuscitation
OHCA: Out-of-hospital cardiac arrest
SBA: Smartphone-based activation

## References

[1] Perkins GD, Handley AJ, Koster RW, Castrén M, Smyth MA, Olasveengen T (2015). European resuscitation council guidelines for resuscitation 2015: section 2. Adult basic life support and automated external defibrillation. Resuscitation.

[2] Hasselqvist-Ax I, Riva G, Herlitz J, Rosenqvist M, Hollenberg J, Nordberg P (2015). Early cardiopulmonary resuscitation in out-of-hospital cardiac arrest. N Engl J Med.

[3] Rajan S, Wissenberg M, Folke F, Hansen SM, Gerds TA, Kragholm K (2016). Association of Bystander Cardiopulmonary Resuscitation and Survival According to ambulance response times after out-of-hospital cardiac arrest. Circulation.

[4] Zijlstra JA, Stieglis R, Riedijk F, Smeekes M, van der Worp WE, Koster RW (2014). Local lay rescuers with AEDs, alerted by text messages, contribute to early defibrillation in a Dutch out-of-hospital cardiac arrest dispatch system. Resuscitation.

[5] Kindness P, Fitzpatrick D, Mellish C, Masthoff J, O'Meara P, McEwan M (2014). An insight into the demands and stressors experienced by community first responders. Journal of Paramedic Practice.

[6] Roberts A, Nimegeer A, Farmer J, Heaney DJ (2014). The experience of community first responders in co-producing rural health care: in the liminal gap between citizen and professional. BMC Health Serv Res.

[7] Phung V-H, Trueman I, Togher F, Orner R, Siriwardena AN (2017). Community first responders and responder schemes in the United Kingdom: systematic scoping review. Scand J Trauma Resusc Emerg Med.

[8] Weir A (2015). Community first responders: improving access to defibrillation in cardiac arrest. Journal of Paramedic Practice.

[9] Heffernan E, Oving I, Barry T, Phung V-H, Siriwardena AN, Masterson S (2019). Factors that motivate individuals to volunteer to be dispatched as first responders in the event of a medical emergency: a systematic review protocol. HRB Open Res.

[10] Timmons S, Vernon-Evans A (2013). Why do people volunteer for community first responder groups?. Emerg Med J.

[11] Hasselqvist-Ax I, Nordberg P, Herlitz J, Svensson L, Jonsson M, Lindqvist J, et al. Dispatch of firefighters and police officers in out-of-hospital cardiac arrest: a nationwide prospective cohort trial using propensity score analysis. J Am Heart Assoc. 2017;6(10):1–8. 10.1161/JAHA.117.005873 PMC5721830 28978527

[12] Scquizzato T, Burkart R, Greif R, Monsieurs KG, Ristagno G, Scapigliati A, Semeraro F. Mobile phone systems to alert citizens as first responders and to locate automated external defibrillators: a European survey. Resuscitation. 2020;151:39–42. 10.1016/j.resuscitation.2020.03.009. 10.1016/j.resuscitation.2020.03.009 32251700

[13] Ming Ng W, De Souza CR, Pek PP, Shahidah N, Ng YY, Arulanandam S, White AE, Leong BS, Ong MEH. myResponder Smartphone Application to Crowdsource Basic Life Support for Out-of-Hospital Cardiac Arrest: The Singapore Experience. Prehosp Emerg Care. 2020:1–9. 10.1080/10903127.2020.1777233. 10.1080/10903127.2020.1777233 32497484

[14] Ringh M, Rosenqvist M, Hollenberg J, Jonsson M, Fredman D, Nordberg P (2015). Mobile-phone dispatch of laypersons for CPR in out-of-hospital cardiac arrest. N Engl J Med.

[15] Andelius L, Malta Hansen C, Lippert FK, Karlsson L, Torp-Pedersen C, Kjær Ersbøll A (2020). Smartphone activation of citizen responders to facilitate defibrillation in out-of-hospital cardiac arrest. J Am Coll Cardiol.

[16] Pijls RWM, Nelemans PJ, Rahel BM, Gorgels APM (2016). A text message alert system for trained volunteers improves out-of-hospital cardiac arrest survival. Resuscitation.

[17] Oving I, Masterson S, Tjelmeland IBM, Jonsson M, Semeraro F, Ringh M (2019). First-response treatment after out-of-hospital cardiac arrest: a survey of current practices across 29 countries in Europe. Scand J Trauma Resusc Emerg Med.

[18] Krammel M, Lobmeyr E, Sulzgruber P, Winnisch M, Weidenauer D, Poppe M (2020). The impact of a high-quality basic life support police-based first responder system on outcome after out-of-hospital cardiac arrest. PLoS One.

[19] Ringh M, Fredman D, Nordberg P, Stark T, Hollenberg J (2011). Mobile phone technology identifies and recruits trained citizens to perform CPR on out-of-hospital cardiac arrest victims prior to ambulance arrival. Resuscitation.

[20] Caputo ML, Muschietti S, Burkart R, Benvenuti C, Conte G, Regoli F (2017). Lay persons alerted by mobile application system initiate earlier cardio-pulmonary resuscitation: a comparison with SMS-based system notification. Resuscitation.

[21] Sarkisian L, Mickley H, Schakow H, Gerke O, Jørgensen G, Larsen ML, Henriksen FL. Global positioning system alerted volunteer first responders arrive before emergency medical services in more than four out of five emergency calls. Resuscitation. 2020;152:170–6. 10.1016/j.resuscitation.2019.12.010. 10.1016/j.resuscitation.2019.12.010 31923531

[22] Saner H, Morger C, Eser P, von Planta M (2013). Dual dispatch early defibrillation in out-of-hospital cardiac arrest in a mixed urban-rural population. Resuscitation.

[23] Hansen CM, Kragholm K, Granger CB, Pearson DA, Tyson C, Monk L (2015). The role of bystanders, first responders, and emergency medical service providers in timely defibrillation and related outcomes after out-of-hospital cardiac arrest: results from a statewide registry. Resuscitation.

[24] Nordberg P, Hollenberg J, Rosenqvist M, Herlitz J, Jonsson M, Järnbert-Petterson H (2014). The implementation of a dual dispatch system in out-of-hospital cardiac arrest is associated with improved short and long term survival. Eur Heart J Acute Cardiovasc Care.

[25] Husain S, Eisenberg M (2013). Police AED programs: a systematic review and meta-analysis. Resuscitation.

[26] Pijls RW, Nelemans PJ, Rahel BM, Gorgels AP (2018). Factors modifying performance of a novel citizen text message alert system in improving survival of out-of-hospital cardiac arrest. Eur Heart J Acute Cardiovasc Care.

[27] Fitch K, Bernstein S, Aguilar MD, Burnand B, LaCalle JR, Lazaro P (2001). The Rand UCLA appropriateness method user's manual: prepared for directorate general XII, European Commission.

[28] Chugh SS, Jui J, Salvucci A (2020). Pivotal role in the community response to cardiac arrest: the smart bystander∗. J Am Coll Cardiol.

[29] Campbell A, Ellington M (2016). Reducing time to first on scene: an ambulance-community first responder scheme. Emerg Med Int.

[30] Smith CM, Wilson MH, Hartley-Sharpe C, Gwinnutt C, Dicker B, Perkins GD (2017). The use of trained volunteers in the response to out-of-hospital cardiac arrest - the GoodSAM experience. Resuscitation.

[31] Phung V-H, Trueman I, Togher F, Ørner R, Siriwardena AN (2018). Perceptions and experiences of community first responders on their role and relationships: qualitative interview study. Scand J Trauma Resusc Emerg Med.

[32] Hasselqvist-Ax I, Nordberg P, Svensson L, Hollenberg J, Joelsson-Alm E (2019). Experiences among firefighters and police officers of responding to out-of-hospital cardiac arrest in a dual dispatch programme in Sweden: an interview study. BMJ Open.

[33] Nordberg P, Jonsson M, Forsberg S, Ringh M, Fredman D, Riva G (2015). The survival benefit of dual dispatch of EMS and fire-fighters in out-of-hospital cardiac arrest may differ depending on population density--a prospective cohort study. Resuscitation.

[34] Holmberg M, Holmberg S, Herlitz J (2001). Factors modifying the effect of bystander cardiopulmonary resuscitation on survival in out-of-hospital cardiac arrest patients in Sweden. Eur Heart J.

[35] Herlitz J, Svensson L, Holmberg S, Angquist K-A, Young M (2005). Efficacy of bystander CPR: intervention by lay people and by health care professionals. Resuscitation.

[36] Nord A, Svensson L, Karlsson T, Claesson A, Herlitz J, Nilsson L (2017). Increased survival from out-of-hospital cardiac arrest when off duty medically educated personnel perform CPR compared with laymen. Resuscitation.

[37] Del Giudice D, Semeraro F, Ristagno G, Picoco C, Cordenons F, Dell'Arciprete O (2019). DAE RespondER: the Emilia Romagna app for a regional “community saving lives” system. Resuscitation.

[38] Harrison-Paul R, Timmons S, van Schalkwyk WD (2006). Training lay-people to use automatic external defibrillators: are all of their needs being met?. Resuscitation.

[39] Zijlstra JA, Beesems SG, de Haan RJ, Koster RW (2015). Psychological impact on dispatched local lay rescuers performing bystander cardiopulmonary resuscitation. Resuscitation.

[40] Scquizzato T, Pallanch O, Belletti A, Frontera A, Cabrini L, Zangrillo A (2020). Enhancing citizens response to out-of-hospital cardiac arrest: a systematic review of mobile-phone systems to alert citizens as first responders. Resuscitation.

[41] Auricchio A, Gianquintieri L, Burkart R, Benvenuti C, Muschietti S, Peluso S (2019). Real-life time and distance covered by lay first responders alerted by means of smartphone-application: implications for early initiation of cardiopulmonary resuscitation and access to automatic external defibrillators. Resuscitation.

[42] Stieglis R, Zijlstra JA, Riedijk F, Smeekes M, van der Worp WE, Koster RW. AED and text message responders density in residential areas for rapid response in out-of-hospital cardiac arrest. Resuscitation. 2020. 10.1016/j.resuscitation.2020.01.031 32045663

[43] McBride R, Ski CF, Thompson DR, Quinn T, Wilson MH (2020). Championing survival: connecting the unknown network of responders to address out-of-hospital cardiac arrest. Scand J Trauma Resusc Emerg Med.

[44] Farmer J, Currie M, Kenny A, Munoz S-A (2015). An exploration of the longer-term impacts of community participation in rural health services design. Soc Sci Med.

